# Advanced glycation end products and their receptors in serum of patients with type 2 diabetes

**DOI:** 10.1038/s41598-021-92630-0

**Published:** 2021-06-24

**Authors:** Diana Indyk, Agnieszka Bronowicka-Szydełko, Andrzej Gamian, Aleksandra Kuzan

**Affiliations:** 1 Department of Medical Biochemistry, Wroclaw Medical University, T. Chałubińskiego 10, 50-368 Wrocław, Poland; 2 Department of Immunology of Infectious Diseases, Hirszfeld Institute of Immunology and Experimental Therapy, Polish Academy of Sciences, Weigla 12, 53-114 Wrocław, Poland

**Keywords:** Biochemistry, Biomarkers, Cardiology, Diseases, Endocrinology, Medical research, Nephrology

## Abstract

Glycation is a non-enzymatic process involving the reaction of reducing sugars or reactive oxoaldehyde with proteins, lipids or nucleic acids, which results in the formation of advanced glycation end products (AGEs). The presented work discusses the glycation process in people with advanced stage of type 1 or type 2 diabetes. The concentration of different AGEs and their receptors for 58 serum samples was determined by ELISA and by spectrofluorimetric methods. In addition to fluorescent low molecular weight and protein-bound AGEs, we have also marked a new class of AGEs: melibiose-derived glycation product (MAGE). Our attention was also focused on the two groups of AGEs receptors: scavenger receptors (SR-A and SR-B) and RAGE. The correlation between the SR-AI scavenging receptors concentration and the fluorescence of AGEs as well as diabetes biological markers: GFR, creatinine contentration and HbA1c was demonstrated. A relationship between the concentration of AGEs and their receptors was also found in serum sample of patients treated with the metformin and aspirin. Furthermore, the concentration of SR-AI scavenger and the fluorescence of total AGEs was significantly lower in treated patients than in non treated patients. AGEs have also been found to contribute to the development of cardiovascular disease, atherosclerosis and diabetic complications, what could be deduced from the correlation of AGEs level and HDL cholesterol or uric acid level. Thus, it was confirmed that AGEs are involved in the pathomechanism of diabetes and other degenerative diseases. Nowadays, it is believed that AGEs due to the long time remaining in the body may be an important diagnostic marker. Their determination may allow monitoring the progression of the disease and the effectiveness of the therapy.

## Introduction

During the heat treatment processes and long food storage, the numerous non-enzymatic chemical reactions take place, called glycation. They also occur in vivo in living organisms between reducing sugars, such as glucose or fructose, and amino acids, peptides or proteins containing a free amino groups. To a lesser extent, glycation also occurs between sugars and phospholipids or nucleic acids^1–3^.

In biological systems glycation takes place in two stages, namely initial and final (advanced). The first stage of glycation is the reversible reaction of an electrophilic carbonyl group or hemiacetal reducing sugar with the free amino group of amino acids (most commonly lysine and arginine). Reaction results in formation of a covalent bond and elimination of water. The resulting product is an unstable Schiff base (imine). This reaction is quite fast, the state of equilibrium is achieved within few hours^4, 5^. It should also be mentioned that individual carbohydrates have a different ability to react with amino groups of peptides or proteins. For example, fructose, often consumed as a replacement for glucose by patients with diabetes, is more reactive in vitro than glucose. Classic substrates for glycation are monosaccharides or dicarbonyls, but glycation products based on the disaccharide melobiose have recently been described^6^. Carbohydrates primarily react with lysine (resulting in N-α-carboxymethyllysine), whereas dicarbons have a higher affinity for arginine^7^. In the next stage lasting several weeks, the Schiff base undergoes an intramolecular rearrangement—the Amadori reaction, which results in a more stable and partly reversible, early glycation product, i.e. ketoamine: 1-amino-1-deoxyketose ^5^. In the presence of oxygen, classic Amadori reaction products are transformed into a substance with a characteristic brownish-yellow color. These changes are often called glycooxidation^8^. Schiff base and Amadori products can irreversibly react with amino acid residues of proteins or peptides to produce protein adducts or cross-linked proteins. Alternatively, these compounds can undergo further Maillard reactions such as oxidation, dehydration, polymerization and numerous transformations with the formation of many reactive oxygen species (ROS)^4^ or dicabonyl compounds (glyoxal, methylglyoxal, ethylglyoxal, 3-deoxyglucosone)^3, 9^. The substances created in this way are strongly reactive. Therefore, they react with amino groups of proteins, leading to the formation of final advanced glycation end products (AGEs)^5^. Adjacent AGE molecules have the ability to connect with each other and with certain proteins, resulting in cross-links that interfere with the function of cells and tissues of the body. AGEs occur in the frequency of one cross-link for several hundred molecules^1^. The resulting final glycation end products exhibit light emission in the range of 420–600 nm after light excitation with a length of 300–420 nm^10^.

Glycation products affect many cells, engage multiple mechanisms, and also use a whole range of receptors. AGEs bind to specific receptors on the surface of various cells, including phagocytes and endothelial cells. There are a few different types of receptors for advanced glycation end products.

### RAGE

RAGE (receptor for advanced glycation end products) is a surface protein with a mass of 45–55 kDa, in the structure of which we can distinguish three fragments with different functions^11^. It consists of a region exposed to the outside of the cell, a single, hydrophobic, transmembrane domain and a highly charged cytosolic tail. It is the first identified transmembrane receptor belonging to the immunoglobulin superfamily, capable of binding a broad repertoire of ligands. sRAGE (soluble form of RAGE) is able to recognize different three-dimensional structures, with not specific amino acid sequences^12^. The sRAGE receptors have been confirmed on the surface of many cell types. They were found on hepatocytes, phagocytes, endothelial cells, vascular walls and smooth muscle, as well as on nervous system cells^13^. RAGE stimulation results in the activation of the nuclear transcription factor (NFκB), followed by the transcription of many pro-inflammatory genes, e.g. adhesion proteins (ICAM-1, VCAM-1), cytokines (IL-1, IL-6, TNF-α) and enzymes related to oxidative stress, for example NADPH oxidase^3, 11, 14^. The affinity of RAGE to AGEs is high, and the complexes between the ligand and the receptor are formed already within nanomolar concentrations^2, 13^. It is postulated that the increase of RAGE receptor expression is stimulated by an increased concentration of advanced glycation end products. Their occurrence leads to constant stimulation of cells, and consequently irreversible tissue damage^1^. This process takes place in pathological states, including immune reactions, diabetic complications, expansion of neoplastic cells^2, 13^.

Another type of AGE binding receptors is called scavenger receptors. It is a superfamily of membrane-associated proteins, defined as class A-J. They bind many ligands, including endogenous proteins and pathogens^15^. Their main task is the endocytosis of modified LDL molecules (Low Density Lipoproteins). Scavenger receptors exhibiting affinity for AGE include SR-AI, SR-BI, CD36, CD68^16^.

### SR-A

SR-A receptors are membrane glycoproteins, in their native form are trimers of spirally entangled polypeptides. The variety of ligands allows SR-A receptors to participate in numerous macrophage functions. The ligands of SR-A receptors include many molecules, mainly polyanions, among others: modified low density lipoproteins (mLDL), such as acLDL and oxidized LDL (oxLDL). In addition, the SR-A receptor binds heat shock proteins, polynucleotides (poly G and poly I), polysaccharides, e.g. dextran sulfate, as well as phospholipids (e.g. phosphatidylserine), AGE^16^. After the complex is formed with the receptors, AGE are removed from the bloodstream^17^.

### SR-B

There are three types of class B scavangers, namely SR-BI (SR-B1), SR-B2 and SR-B3. SR-B receptors are surface glycoproteins involved in lipid metabolism. SR-BI is known mainly as high density lipoprotein (HDL) receptors, which mediate the selective uptake of cholesterol esters in the cell^16^. SR-BI is expressed in the liver, heart, brain and macrophages, where they occur in increased quantity^15^. The SR-BI receptor for liver and steroidal tissues provides cholesterol esters for the purpose of their removal from the body or the synthesis of steroid hormones. SR-BI may also facilitate the outflow of cholesterol from cells, e.g. from macrophages. The SR-BI receptor is involved in various processes, including platelet aggregation, oxidative stress, apoptosis and activation of nitric oxide synthase. Hepatic SR-BI plays a key role in the clearance of HDL lipoproteins from plasma and consequently, a decrease in HDL cholesterol in plasma. Expression of this class of receptors in the liver is a key positive regulator of the rate of return transport of cholesterol from macrophages to faeces. Consistent with this concept, it was shown that SR-BI may play antiatherosclerotic function in mice^18^, although it is also reported that AGEs can promote macrophage expression of SR-BI, what may induce lipid accumulation and foam cell formation, stimulating the progression of atherosclerosis^19^.

The scavenger receptor SR-B2 is known also as CD36, SCARB3, fatty-acid translocator (FAT) and glycoprotein 4 (GPIV)^20^. Its ligands, in addition to native and oxidized lipids also includes advanced glycation products, hydrophobic peptides, thrombospondin-1, collagen, amyloid B and apoptotic cell fragments^20, 21^. The third protein in this family—SR-B3, also called Lysosomal Integral Membrane Protein 2 (LIMP-2) is involved in the transport of cholesterol and β-glucocerebrosidase beetwen endoplasmic reticulum and lysosomes^22, 23^.

The aim of the project was to check whether there is a close relationship between the occurrence of diabetes, diabetic complications, atherosclerosis and hypertension and the increased content of advanced glycation end products and their receptors in the blood of patients. Our attention was also focused on checking the effectiveness of diabetes treatment with a number of drugs considered to be AGE inhibitors. The performed statistical analysis revealed a significant relationship between the fluorescence and concentration of AGEs and their receptors, and the use of this group of drugs by patients.

## Results

The quantitative analysis of AGE and it’s receptors content allowed to conclude that the analyzed material consists on average of 0.576 ± 0.050 [mg/ml] MAGE (competition ELISA, proteinase K digested samples); 1.161 ± 0.0234 [mg/ml] MAGE (indirect ELISA, proteinase K digested samples); 0.216 ± 0.040 [µg/ml] SR-BI; 0.206 ± 0.016 [µg/ml] SR-A; 11353 ± 2147 [pg/ml] sRAGE. The values given are the mean of the results obtained during the experiments and the standard errors of the mean (Mean ± SE).

The concentration of advanced glycation end products was higher for the patients sera digested with proteinase K than those that were not digested in the vast majority of cases. The reason for this result is most likely the disclosure with an enzyme of epitopes, inaccessible to antibodies inside the molecule. However, it is worth adding that the cross-links characteristic of glycation are not fully susceptible to enzymatic activity ^24^. In addition, the results of both analyzes correlate quite closely with each other (r= 0.7517, *p*<0.0001), it means that it is possible to simplify test in order to obtain reliable results without proteinase K.

Below is a description of the correlations between the analyzed parameters. We omitted the statistically insignificant and interpreted the statistically significant ones.

**Figure 1:**
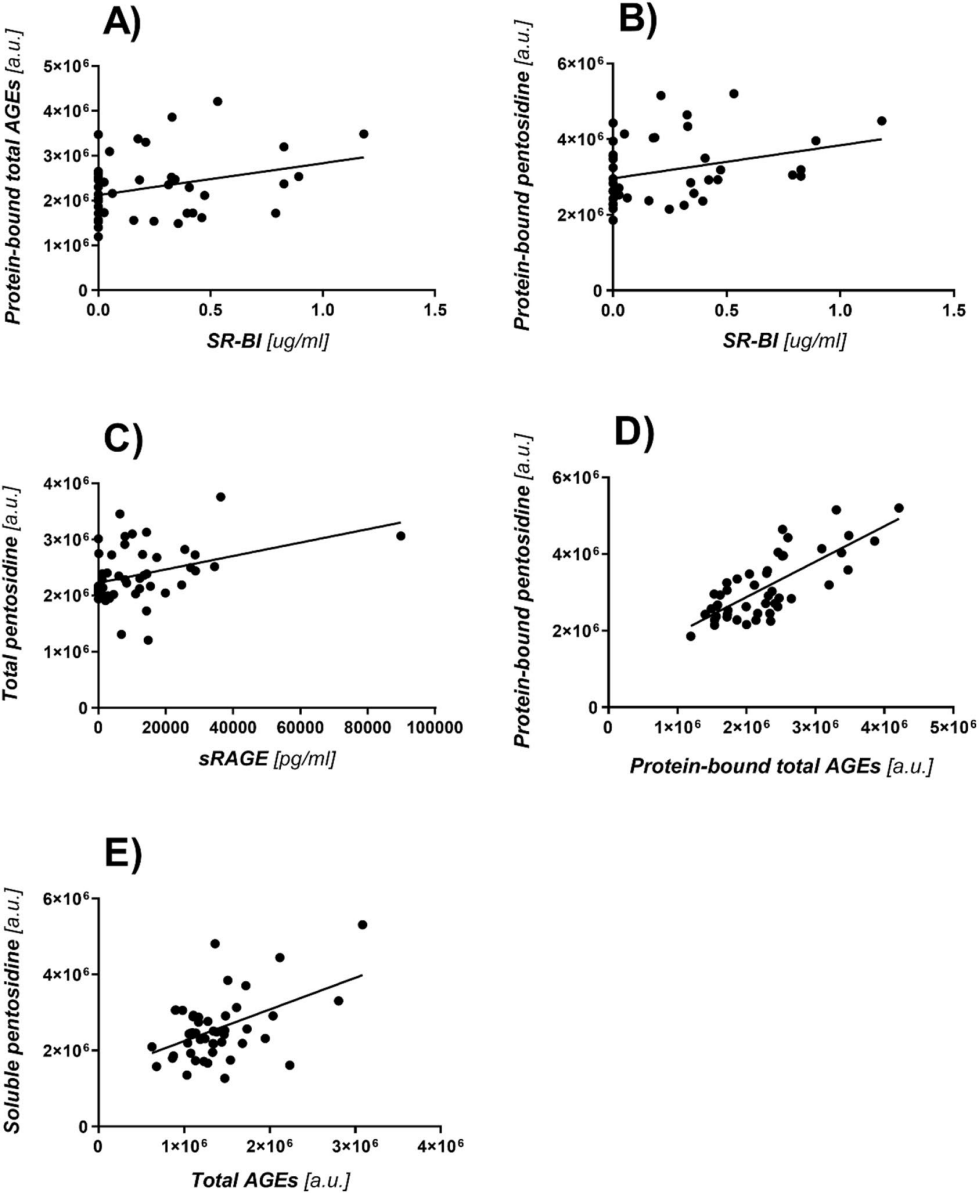
(**A**) Graph of the correlation between the concentration of SRBI receptors and total AGEs bound to proteins, (*p* = 0.0493, r = 0.3052). (**B**) Graph of the correlation between SR-BI and pentosidine bound to proteins, (*p* = 0.0481, r = 0.3068). (**C**) Graph of the correlation between sRAGE and pentosidine measured in the whole sample, (*p* = 0.0094, r = 0.3675). (**D**) Graph of correlation between protein-bound total AGEs and protein-bound pentosidine, (*p* < 0.0001, r = 0.761). (**E**) Graph of the correlation of AGEs measured in the whole sample and soluble pentosidine, (*p* = 0.0006, r = 0.480).

The existence of a correlation between the concentration and fluorescence of advanced glycation end products and their receptors: SR-BI and sRAGE was found (Fig. 1). The results clearly show that the more AGEs, the higher the concentration of interacting receptors in the blood serum of patients suffering from diabetes.

**Figure 2:**
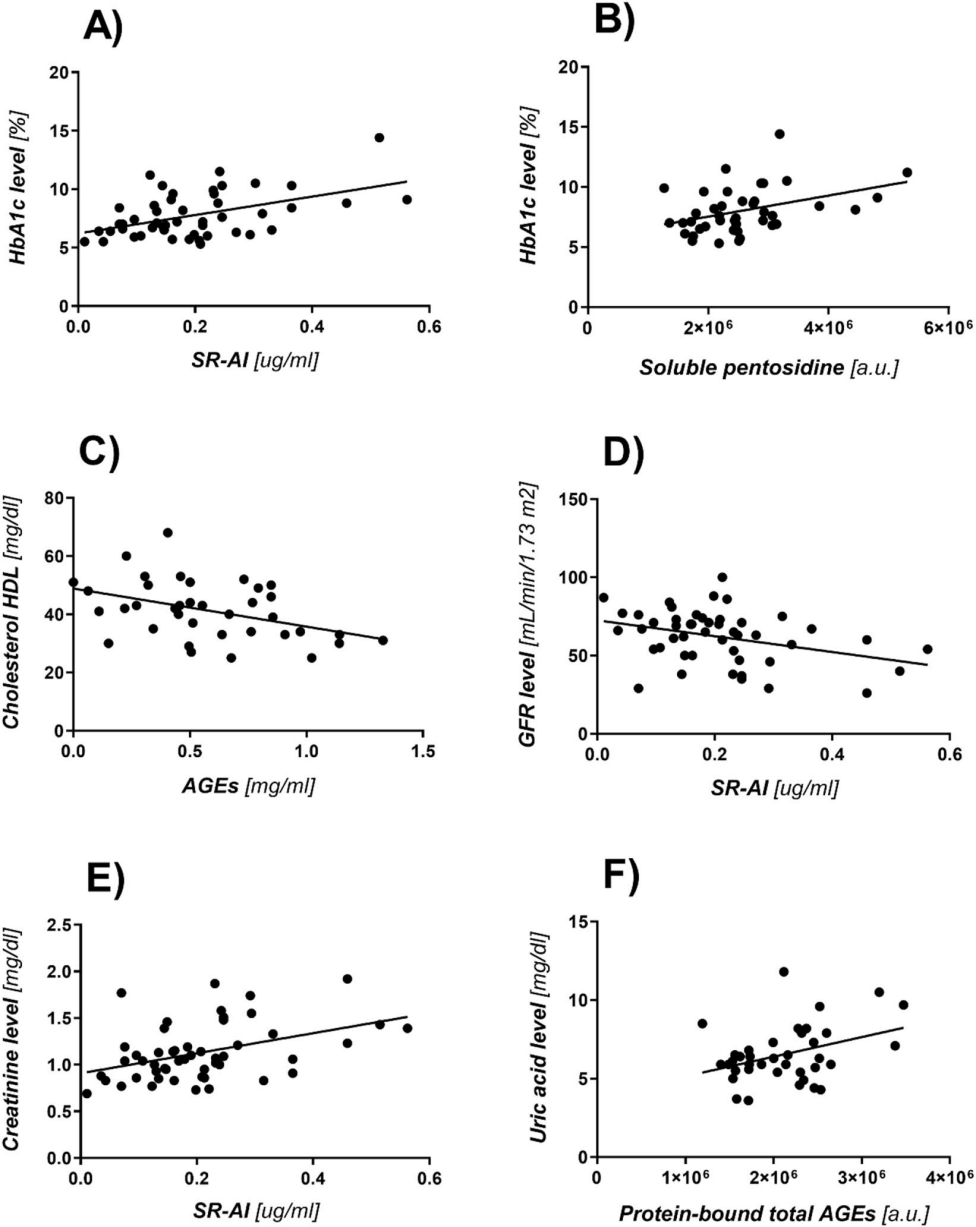
(**A**) Graph of the correlation between the level of glycated hemoglobin HbA1c and the concentration of the SR-AI scavenger receptor in the samples. (**B**) Graph of the correlation between the level of glycated hemoglobin HbA1c and fluorescence of soluble pentosidine. (**C**) Graph of the correlation between the concentration of HDL and AGEs content in samples digested with proteinase K (the competitive ELISA test). Graph of the correlation between the levels of GFR. (**D**) and creatinine. (**E**) and the concentration of the SR-AI receptor in the serum samples. (**F**) Graph of the correlation between the level of uric acid and the fluorescence value of protein-bound total AGEs in the serum samples.

Statistical analysis showed that there is a number of correlations between the numerical blood parameters of patients and the results of experiments:

The level of glycated hemoglobin HbA1c and the SR-AI content in samples (*p* = 0.0005, r = 0.4828; Fig. 2A),

The level of glycated hemoglobin HbA1c and fluorescence value of soluble pentosidine (*p* = 0.0098, r = 0.3941; Fig. 2B),

The concentration of HDL and MAGE content in the samples digested with proteinase K (the competitive ELISA test) (*p* = 0.0093, r =− 0.4220; Fig. 2C),

Systolic blood pressure RRs and MAGE content in the samples non-digested with proteinase K (the competitive ELISA test), (*p* = 0.0463, r = 0.4105),

Diastolic blood pressure RRr and MAGE content in the samples digested with proteinase K (the competitive ELISA test) (*p* = 0.0010, r = 0.5185),

GFR filtration rate and the SR-AI content (*p* = 0.01320, r = − 0.3553; Fig. 2D),

Creatinine level and the SR-AI content in the samples (*p* = 0.0023, r = 0.4292; Fig. 2E),

Uric acid level and fluorescence value of protein-bound total AGEs (*p* = 0.0221, r = 0.3726; Fig. 2F).

**Figure 3:**
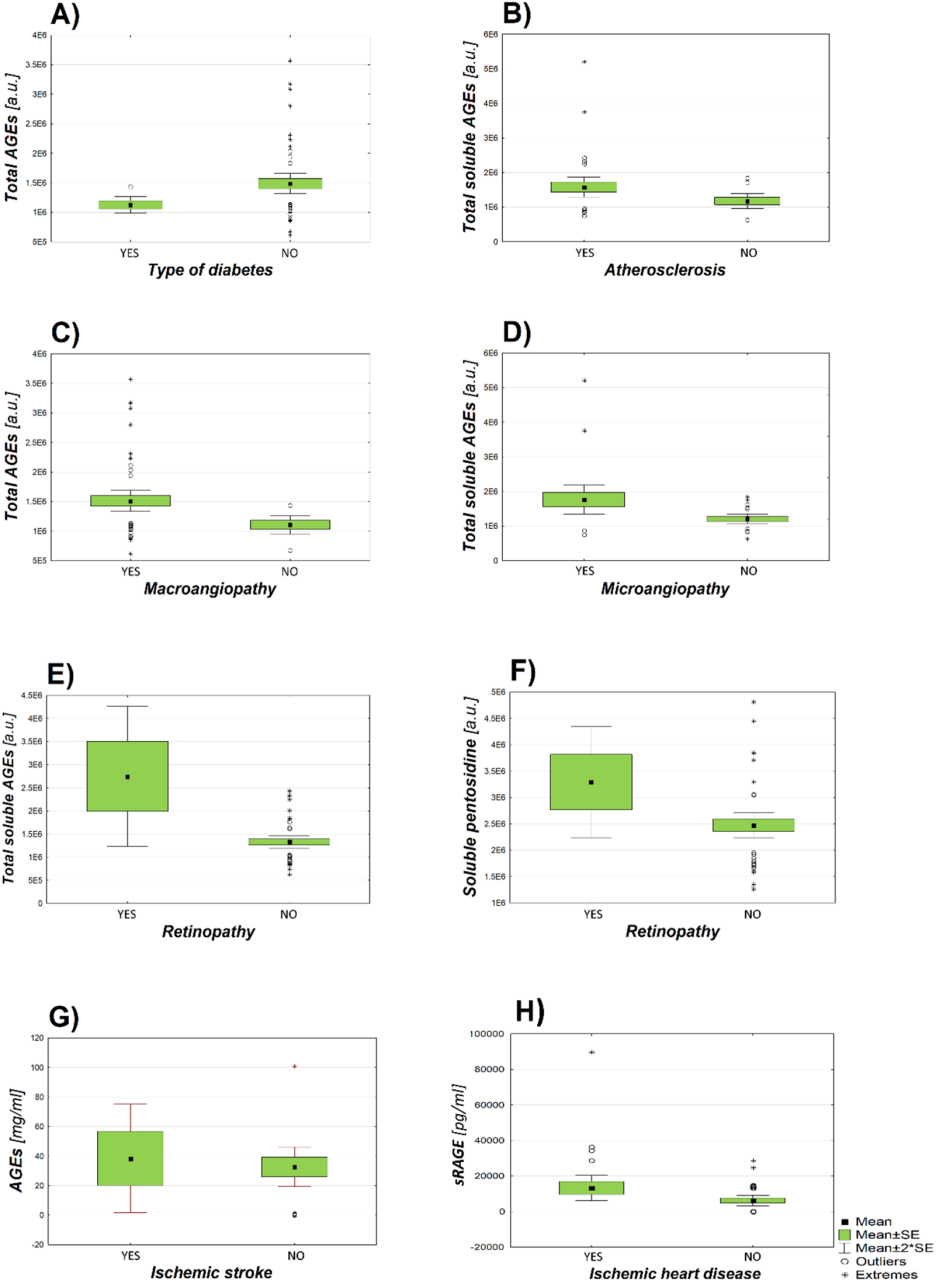
Graphs of the dependence of AGEs fluorescence or content of sRAGE in the samples and occurrence diabetic complications in patients. The numbers given are the mean value of the results and the standard error of the mean (Mean ± SE). (**A**) The dependence of total AGEs fluorescence on the type of diabetes; (number of subjects = 49; Type 1 (1,167,393 ± 70,632 a.u.), Type 2 (1,517,352 ± 98,785 a.u.)). (**B**) The dependence of total soluble AGEs fluorescence on the occurrence of atherosclerosis; (n = 47; YES (1,581,783 ± 145,154 a.u.), NO (1,179,192 ± 107,389 a.u.)). (**C**) The dependence of total AGEs fluorescence on the occurrence of macroangiopathy; (n = 58; YES (1,511,867 ± 619,228 a.u.), NO (1,105,011 ± 228,314 a.u.)). (**D**) The dependence of total soluble AGEs fluorescence on the occurrence of microangiopathy; (n = 47; YES (1,764,035 ± 207,484 a.u.), NO (1,205,829 ± 68,639 a.u.)). (**E**) The dependence of total soluble AGEs fluorescence on the occurrence of retinopathy; (n = 47; YES (2,749,771 ± 757,703 a.u.), NO (1,327,710 ± 66,108 a.u.)). (**F**) The dependence of soluble pentosidine fluorescence on the occurrence of retinopathy; (n = 47; YES (3,320,508 ± 527,192 a.u.), NO (3,069,839 ± 117,568 a.u.)). (**G**) The dependence of AGEs concentration determined in the competition ELISA test on the occurrence of ischemic stroke; (n = 39; YES (0.9252 ± 0.0687 mg/ml), NO (0.5244 ± 0.0517 mg/ml)). (**H**) The dependence of the concentration of sRAGE receptors on the occurrence of ischemic heart disease; (n = 49; YES (15,467 ± 3967 pg/ml), NO (7404 ± 1628 pg/ml)).

The relationship between individual blood parameters and the results on AGEs and it’s receptors experiments was assessed. Statistical analysis showed that there are statistically significant relationships between the results of experiments and the number of different diseases:

Type of diabetes and fluorescence value of total AGEs (*p* = 0.008; Fig. 3A),

Atherosclerosis and fluorescence value of total soluble AGEs (*p* = 0.031; Fig. 3B),

Macroangiopathy and fluorescence value of total AGEs (*p* = 0.001; Fig. 3C),

Microangiopathy and fluorescence value of total soluble AGEs (*p* = 0.017; Fig. 3D),

Retinopathy and fluorescence value of total soluble AGEs (*p* = 0.003; Fig. 3E),

Retinopathy and fluorescence value of soluble pentosidine (*p* = 0.013; Fig. 3F),

Ischemic stroke and MAGE content in the samples digested with proteinase K (competitive ELISA test) (*p* =0.006; Fig. 3G),

Ischemic heart disease and sRAGE content in the samples (*p* = 0.042; Fig. 3H).

All statistically significant relationships are presented in Table 1. Some important relationships are presented graphically (Fig. 3).

**Table 1:** The dependence of basic information about the health of patients on the concentration and fluorescence of AGEs and the concentration of AGE receptors. The statistically significant values are shown in red. PL—significance level for Levene’s test; PV – significance level for tke student’s t-test from the analysis of equality of variance; PNV – significance level for the students’s t-test of inequality of variance.

	Mean concentration/fluorescence value (± Std. Error Mean)	PL	PV	PNV
Diabetes yes (n = 35) no (n = 4)	MAGE (competition ELISA test) [mg/ml] 0.602 ± 0.053 0.351 ± 0.138	0.423	0.134	0.165
Type of diabetes Type 1 (n = 5) Type 2 (n = 44)	Total AGEs [a.u.] 1,167,393 ± 70,632 1,517,352 ± 98,785	0.076	0.224	0.008
Atherosclerosis yes (n = 35) no (n = 12)	Total soluble AGEs [a.u.] 1,581,783 ± 145,154 1,179,192 ± 107,389	0.221	0.124	0.031
Insulin therapy yes (n = 17) no (n = 31)	MAGE (indirect ELISA test) [mg/ml] 0.954 ± 0.306 1.275 ± 0.327	0.165	0.552	0.477
Hypertension yes (n = 42) no (n = 2)	MAGE (indirect ELISA test) [mg/ml] 1.212 ± 0.244 0.0 ± 0.0	0.042	0.311	0.001
Hyperlipidemia yes (n = 43) no (n = 5)	MAGE (indirect ELISA test) [mg/ml] 1.287 ± 0.257 0.083 ± 0.061	0.003	0.121	0.001
Smoking yes (n = 8) no (n = 39)	Protein-bound pentosidine [a.u.] 3,983,087 ± 385,595 3,008,319 ± 118,728	0.033	0.036	0.131
Macroangiopathy yes (n = 49) no (n = 9)	Total AGEs [a.u.] 1,511,867 ± 619,228 1,105,011 ± 228,314	0.060	0.058	0.001
Microangiopathy yes (n = 23) no (n = 24)	Total soluble AGEs [a.u.] 1,764,035 ± 207,484 1,205,829 ± 68,639	0.017	0.013	0.017
Retinopathy yes ( n = 5) no (n = 42)	Total soluble AGEs [a.u.] 2,749,771 ± 757,703 1,327,710 ± 66,108 Soluble pentosidine [a.u.] 3,320,508 ± 527,192 3,069,839 ± 117,568	0.114 0.141	0.003 0.013	0.021 0.056
Polyneuropathy yes (n = 23) no (n = 24)	MAGE (indirect ELISA test) [mg/ml] 1.257 ± 0.321 1.065 ± 0.353	0.589	0.689	0.689
Ischemic heart disease yes (n = 24) no (n = 25)	sRAGE [pg/ml] 15,467 ± 3967 7404 ± 1628	0.042	0.062	0.070
Ischemic stroke yes (n = 5) no (n = 34)	MAGE (competitive ELISA test) [mg/ml] 0.9252 ± 0.0687 0.5244 ± 0.0517	0.224	0.006	0.001

**Figure 4:**
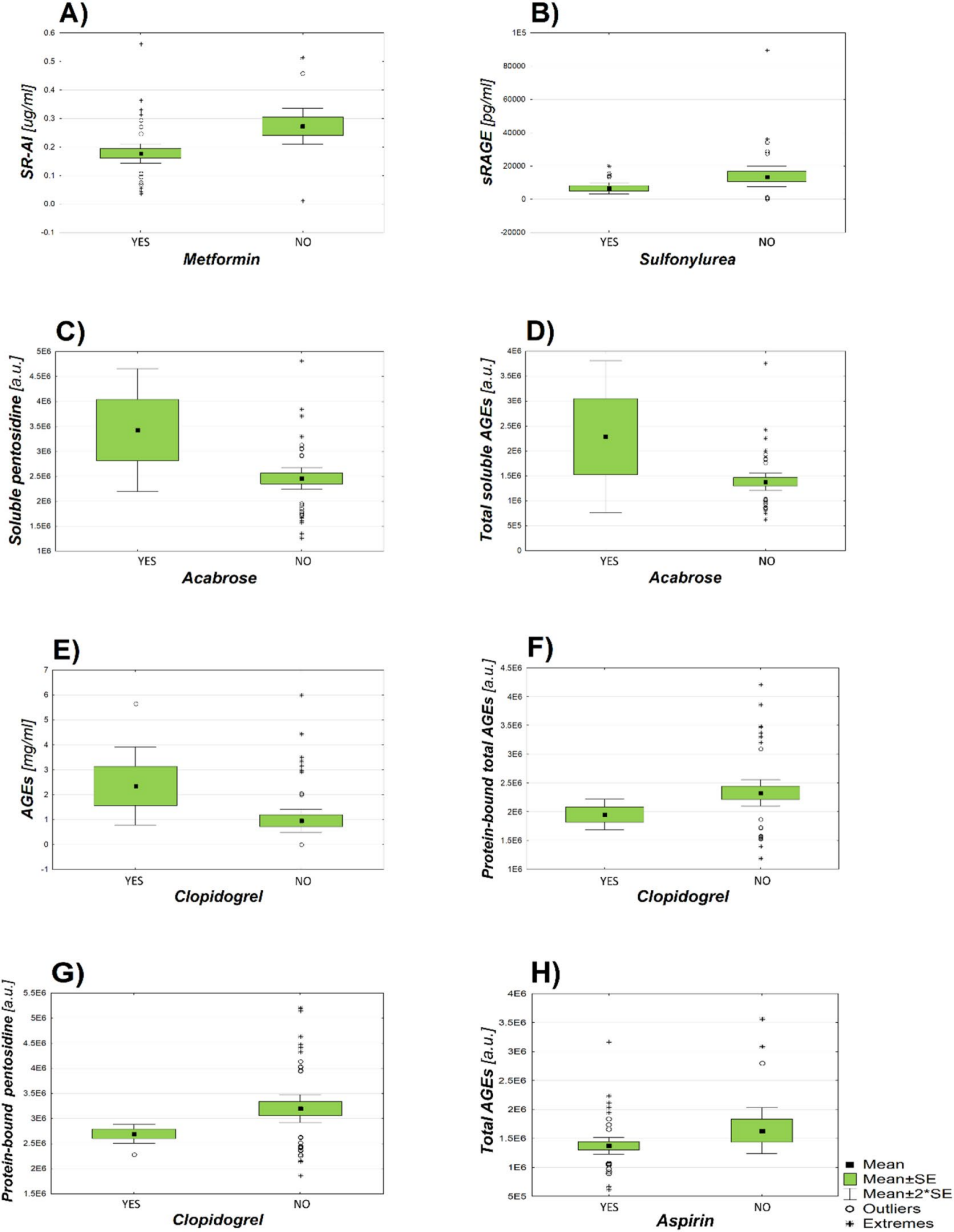
Graphs of dependence of the intake of AGEs inhibitors by patients and the content of AGEs and their receptors in the blood serum. The values given below are the mean of the results and the standard errors of these means (Mean ± SE). (**A**) The dependence of the concentration of SR-AI receptors in the blood serum of patients on the intake of metformin; (number of subjects = 54; YES (0.1773 ± 0.017 µg/ml), NO (0.273 ± 0.032 µg/ml)). (**B**) Dependence of the concentration of sRAGE receptors on the intake of sulphnylurea; (n = 49; YES (6577 ± 1699 pg/ml), NO (13,669 ± 3052 pg/ml)). (**C**) Dependence of soluble pentosidine fluorescence on the intake of acabrosis; (n = 46; YES (2,749,066 ± 232,013 a.u.), NO (3,167,709 ± 132,824 a.u.)). (**D**) Dependence of total soluble AGEs fluorescence on acarbose intake; (n = 47; YES (2,285,230 ± 762,302 a.u.), NO (1,383,013 ± 86,164 a.u.)). (**E**) Dependence of AGEs concentration on the intake of clopidogrel; (n = 48; YES ( 2.351 ± 0.783 mg/ml), NO (0.9579 ± 0.233 mg/ml)). (**F**) Dependence of fluorescence of protein-bound low-molecular AGEs on clopidogrel intake; (n = 47; YES (2,694,944 ± 94,577 a.u.), NO (3,198,113 ± 139,294 a.u.)). (**G**) Dependence of total protein-bound AGEs fluorescence on clopidogrel intake; (n = 47; YES (1,950,442 ± 132,149 a.u.), NO (2,326,301 ± 113,815 a.u.)). (**H**) Dependence of total AGEs fluorescence on aspirin intake; (n = 58; YES (1,372,031 ± 71,806 a.u.), NO (1,633,724 ± 199,279 a.u.)).

A number of drugs are known to be AGEs inhibitors^25^. These small molecules have the ability to break preformed AGEs or inhibit AGEs formation. They have enormous therapeutic potential in the treatment of diabetic cardiovascular disease ^25^. In our research, we analyzed different types of advanced glycation end product inhibitors. The effect of taking metformin, acabrosis, cholinergic drugs, aspirin, clopidogrel and sulfonylurea on the content of AGEs and their receptors in the blood serum of patients was examined. Important relationships are presented graphically (Fig. 4). A number of key relationships were shown below:

Taking metformin and the content of SR-AI (*p* = 0.006; Fig. 4A )

Taking sulfonylurea and the content of sRAGE (*p* = 0.048; Fig. 4B)

Taking acabrose and fluorescence value of soluble pentosidine (*p* = 0.013; Fig. 4C)

Taking acabrose and fluorescence value of total soluble AGEs (*p* = 0.013; Fig. 4D)

Taking clopidogrel and the content of MAGE in the samples digested with proteinase K (indirect ELISA test) (*p* = 0.036; Fig. 4E)

Taking clopidogrel and the fluorescence value of protein-bound total AGEs (*p* = 0.046; Fig. 4F)

Taking clopidogrel and the fluorescence value of protein-bound pentosidine (*p* = 0.004; Fig. 4G)

Takinig aspirin and fluorescence value of total AGEs (*p* = 0.007; Fig. 4H)

## Discusion

Diabetes is caused by abnormal glucose levels or inappropriate tolerance, accompanied by an insufficient insulin response. Unfortunately, the incidence of diabetes is constantly increasing and is one of the leading causes of death for both women and men. Its occurrence is directly related to the increasing prevalence of overweight or obesity and may lead to a range of diabetic complications and even premature death^26^. This effect is of particular importance in connection with the COVID-19 epidemic, because diabetes is one of the risk factors for the acute course of COVID-19^27^. That is why it is so important to conduct research on the factors influencing the development of diabetes. They have a chance to contribute to faster diagnosis and effectively improve the effectiveness of the treatment of this disease.

There is more and more evidence that the interaction of AGEs and their receptors (RAGE) causes oxidative stress, inflammatory reactions, the formation of areas of calcification and the formation of blood clots in the walls of the arteries. In this way, advanced glycation products are involved in the aging of blood vessels and their numerous damages. These observations suggest that the combination of AGEs with their receptors is a new therapeutic target in the prevention of vascular complications in diabetes^2, 3, 28^.

HbA1c is considered to be an important marker of long-term glycemic control and a reliable biomarker for the diagnosis and prognosis of diabetes. HbA1c is formed in the glycation process, is a product of Amadori rearrangement, and accumulates in the red blood cell. Its high level proves not only chronic hyperglycemia, but also correlates well with its risk. Moreover, it is considered to be an independent risk factor for coronary heart disease and stroke, obesity-related metabolic disorders and carotid atherosclerosis even in non diabetic individuals^29, 30^. By comparing the results obtained during the experiments with the information on the level of glycated hemoglobin in individual patients, a relationship was demonstrated between the SR-AI scavenger receptor and the level of HbA1c (r=0.4828, *p*=0.0005). It is worth adding that the family of scavenging receptors is involved not only in atherosclerosis, but also in inflammation, immune response and, most importantly, in other chronic diseases such as diabetes. The relationship between the level of glycated hemoglobin in serum samples and the fluorescence of soluble pentosidine was also confirmed (r=0.3941, *p*=0.0098). A positive correlation between these two parameters was also demonstrated by Koyama et al. A high level of HbA1c is associated with a high concentration of SR-AI scavenging receptors. Their task is to remove from the body AGEs transformed from early glycation products as a result of a series of non-enzymatic transformations^31^. However, no correlation was found between the concentration of AGEs or their receptors and the level of fasting glucose. This confirms that the level of glycated hemoglobin in the blood of patients may be more reliable marker of diabetes than blood sugar levels.

Creatinie is considered as a “biomarker for a risk factor” i.e. a marker of risk for nephropathy, diabetic retinopathy, and other vascular complications.The results of the conducted research also suggest that SR-A can be considered as a prognostic factor for diabetes^32^.

Stiffening of arterial vessels and increased blood pressure are the main risk factors for cardiovascular disease in the elderly. According to experimental data, the formation of advanced glycation end products is associated with the stiffness of blood vessels and thus an increase of blood pressure^33^. AGEs act directly and through receptors to change the function of many intracellular and extracellular proteins, including metabolic enzymes and calcium channels. This causes endothelial dysfunction, inflammation, and oxidative stress. We compared the concentration of MAGE with measurements of systolic (RRs) and diastolic (RRr) pressures using univariate linear regression. We obtained results clearly show that MAGE levels were significantly higher in people with hypertension than in people with normal blood pressure (r=0.5185, *p*=0.001). Vasdev et al. also found that there is a strong correlation between the concentration of AGEs in plasma and the increase in systolic and diastolic blood pressure. It is worth noting that the pathological role of AGEs is indirectly confirmed by studies showing that therapies reducing insulin resistance are effective in relieving oxidative stress, high blood pressure and atherosclerotic vascular changes^34^.

Statistical analysis showed a negative correlation between the level of HDL cholesterol (high-density lipoprotein) and the concentration of MAGE (r = −0.4220, *p* = 0.0093). The more MAGEs in the blood serum of patients, the lower the level of HDL, i.e. "good" cholesterol. There was no statistically significant correlation between AGEs concentration or total cholesterol and LDL (low-density lipoprotein). Nevertheless, the negative correlation of MAGE with HDL and other markers of atherosclerosis suggests that MAGE may be a marker of arteriosclerosis in diabetic patients. For classic AGEs this has been stated by numerous studies^2, 19, 35^, we state this for the first time for AGEs based on melobiose.

Glomerular filtration rate depends on the age, sex, race and body weight of the tested person.

It is considered an important indicator of the excretory function of the kidneys. Early studies of GFR levels and estimated glomerular filtration rate (eGFR) calculated from serum creatinine level, allow to detect chronic renal failure, which is an increasingly serious health problem for people around the world. Chronic failure of this organ may be caused by ischemia, nephritis, glomerulonephritis or diabetic nephropathy, which occur in people with undiagnosed or improperly treated diabetes^36^. The results obtained during the experiments were compared with information on the level of GFR and creatinine in individual patients. There was a negative correlation between the concentration of the scavenger SR-AI receptor and the level of these two parameters (GFR, creatinine) in the patients serum samples (r = −0.3552 *p* = 0.01320; r = 0.4292 *p* = 0.0023). The result of the analysis is predictable and confirmed in other studies^37^. Advanced glycation end products and their receptors are involved in the pathogenesis of kidney disease. Although their relationship to the level of kidney function has not been well characterized, we know that AGEs are responsible for a number of health conditions. The kidney is both the target and the "culprit" of AGEs. Inhibition of this organ function is associated with higher concentration of circulating AGEs through increased formation as well as decreased clearance. On the other hand, AGEs are involved in renal pathophysiology, degenerative structural changes in the renal parenchyma, including tubular atrophy, which is most prominent in diabetic nephropathy^10, 38^.

Uric acid is important in the diagnosis of kidney diseases and gout as one of the parameters in biochemical tests. It is a substance with pro-oxidative properties, a key extracellular antioxidant with detrimental effects at very high serum concentrations. High uric acid concentration in patients serum is a risk factor for atherosclerosis and hypertension. Moreover, it can be a valuable marker of oxidative stress—important from the point of view of cardiovascular diseases^39^.

A positive correlation was demonstrated after comparing the results of AGEs fluorescence with the level of uric acid in the serum samples of patients (r = 0.3726, *p* = 0.0212). This confirms that with the increase of AGEs concentration, the level of uric acid increases in the blood, which is also confirmed by the research conducted by Koyama et al.^40^. We can conclude that glycation products are involved in the development of microangiopathic complications of diabetes, i.e. diabetic nephropathy, because they probably accumulate in the glomeruli and in the blood vessels of the kidneys.

Many studies have assessed the involvement of AGEs in the development of type 2 diabetes, in particular in the development of insulin resistance, β-cell dysfunction, and their role in diabetic complications^37, 41^. Studies indicate a relationship between an increase in the concentration of advanced glycation end products and the occurrence of diabetes^31, 41^. Here it is presented a relationship between the type of diabetes and the level of AGEs in the blood serum. Patients with type 2 diabetes had significantly higher AGEs fluorescence and a much higher concentration of glycation end products and their receptors: SR-BI and sRAGE, compared to the group of patients with type 1 diabetes.

Hyperlipidaemia, hypertension, and hyperglycaemia play an important role in the development of atherosclerosis in people with diabetes. In hyperglycemia and often accompanying hyperlipidemia, changes in glucose and lipid metabolism enhance glycation processes. This leads to the formation of advanced glycation end products, which contributes to the development of oxidative stress, increased blood pressure, endothelial dysfunction, development of atherosclerotic plaques and increased post-glycation destruction of cells and tissues^42^. Zucker studies on obesity in experimental rats based on the insulin resistance model with pyridoxamine (an inhibitor of AGE formation) showed a significant reduction in the characteristic hyperlipidemia and a lower amount of advanced lipoxidation end products (ALE), indicating an association between AGE and lipids^34^. Our studies confirmed the significant connection of advanced glycation end products with the development of dyslipidemia and atherosclerosis. There was a correlation between these diseases and an increase in AGEs in the blood serum (*p* = 0.001, *p* = 0.031). This relationship has also been demonstrated by Raposeiras-Roubin et al.^43^.

Many experiments have shown a correlation between the levels of AGEs and their receptors in the serum and the development and severity of heart failure. For example, the experiment performed by Raposeiras-Roubin et al.^44^ have shown that increased serum sRAGE levels are associated with increased heart failure, especially in patients with ischemic heart disease. In the work of Ikeda et al.^45^ the correlation was found between a high concentration of low-molecular AGEs and acute ischemic stroke. Namely, it has been shown that patients with ischemic stroke have a higher concentration of MAGE than patients who didn't have a stroke (*p*=0.006). Moreover, it has been shown that patients suffering from ischemic heart disease have a much higher concentration of sRAGE receptors (*p* = 0.042). Research results indicate that advanced glycation end products and their receptors may constitute a new therapeutic target in the treatment of cardiovascular diseases, especially heart failure and stroke.

The study showed a relationship between the high fluorescence of soluble total AGEs and the occurrence of macro- and microangiopathy (*p*=0.001, *p*=0.017). The connection between the high fluorescence of total and low molecular mass AGEs associated with proteins was also confirmed. An association was also demonstrated between the occurrence of retinopathy in patients and

a high degree of fluorescence of soluble total and low-molecular AGEs (*p* = 0.003, *p* = 0.013). The results obtained from the experiments confirm the fact that the high concentration of AGEs plays an important role in the pathogenesis of diabetic complications. Numerous studies indicate that a large amount of glycation end products affects the functional properties of the extracellular matrix, intracellular signal transduction and protein function, consequently leading to dysfunction of many organs^8, 31^.

Research proves that free radicals play an important role in the pathogenesis of civilization diseases, which contribute to the exposure of the body to oxidative stress. Their source is the influence of environmental xenobiotics, contained i.e. in tobacco smoke^1^. It is one of the factors that induce the formation of large amounts of free radicals, which the body is unable to detoxify, and consequently lead to numerous damage to cells and tissues. Research shows that tobacco smoke is one of the sources of environmental exposure to glycation end products^46^. Our statistical analysis of the relationship between smoking patients and the concentration/fluorescence of AGEs and their receptors determined during the experiments confirms this result. It was shown that in the majority of analyzes, the concentration and fluorescence of AGEs in the group of smoking patients was higher than in the group of non-smokers.

A number of drugs considered to be AGE inhibitors have been developed. These include, i.e. anti-inflammatory drugs with anti-glycation properties (diclofenac, acetylsalicylic acid), metabolically active drugs and vitamins with anti-AGE properties (pyridoxamine, metformin), antioxidants and free radical scavengers (herbal extracts rich in flavonoids, carnosine) and AGE inhibitors with chelating properties (pyridoxamine, acarbose). The compounds preventing glycation also include substances inhibiting excessive aggregation of platelets (clopidogrel), and a group of compounds capable of producing endogenous insulin, e.g. sulfonylurea^47, 48^. In addition to reducing hepatic glucose production and increasing cell sensitivity to insulin, many studies have shown that metformin administration is beneficial in reducing vascular risk associated with diabetes. This occurs by inhibiting glycation and intermediates ^14^. Research by Hyun et al. shows that the expression of the scavenger receptors CD36 and SR-A was impaired by metformin in macrophages of obese mice. These results suggest that metformin may impair inflammatory responses by inhibiting TNF-α production and reducing the expression of scavenger receptors^49^. Our studies have shown that the concentration of SR-AI scavenging receptors responsible for the removal of advanced glycation-modified end products of proteins is significantly reduced in patients treated with metformin (*p* = 0.006), which confirms the efficiency of the treatment of diabetes with this compound.

Acetylsalicylic acid has anticoagulant and anti-inflammatory properties, therefore it is used in the prevention of cardiovascular diseases. Numerous studies show a relationship between taking aspirin and decreased tissue AGEs concentration, because aspirin inhibits glycation in vitro. It may work by chelating metal ions and by scavenging oxygen radicals^48, 50^. Our analysis showed a significant relationship between aspirin treatment and a lower fluorescence of total AGEs (*p* = 0.007). The result is expected and confirms the effectiveness of glycation inhibition with this compound.

The effect of another anti-AGE compounds—clopidogrel, which is a drug that inhibits platelet aggregation^47^ and sulphonylurea which stimulates the synthesis of endogenous insulin^51^ was investigated. A lower fluorescence of protein-bound AGE and protein-bound pentosidine is observed in patients taking clopidogrel. There was also

a significant association between the intake of sulphonylurea and a higher concentration of sRAGE in the blood serum of patients (*p* = 0.048). sRAGE binds plasma AGEs by competing with endothelial RAGE, which protects the arterial walls from oxidative stress. From this we conclude that sulphonylurea counteracts the effects of AGE formation while clopidogrel probably inhibits the formation of fluorescent AGEs.

Performing the statistical analysis, a significant correlation was found between the intake of acarbose by patients and the fluorescence of total soluble AGEs (*p*=0.013) or soluble pentosidine (*p*=0.013). Acarbose is an inhibitor of α-glucosidase, which delays digestion and absorption of carbohydrates, thus reducing the postprandial rise in blood glucose. It has also been found that acarbose inhibits the intima layer bold in the artery and reduces the existence of cardiovascular diseases^52^.

The relationship between taking the above-mentioned drugs (clopidogrel, sulfonylureas, acarbose) and increased fluorescence of AGEs and a higher concentration of their receptors (SR-BI, sRAGE) is expected. Many studies, including those carried out by Tsunosue et al. and Urios et al.^50, 53^ show that treatment with these drugs reduces the amount of AGEs and their receptors in the blood serum. More extensive research is needed to determine the effect of these drugs on a specific AGE. It is worth explaining why clopidogrel has

a different effect on protein-bound AGEs than on total AGEs.

## Summary and conclusions

To our knowledge, this is the first publication that describes different types and fractions of advanced glycation products and as many as 3 types of receptors for AGEs. Particularly valuable is the determination of the MAGE content and the description of observations related to this new class of glycation products in the diabetic population.

Many results indicate that long-term consumption of highly processed food is associated with the emergence of metabolic syndrome and insulin resistance. As a result, the probability of inflammation and cardiovascular complications that induce type 2 diabetes is increased^3^. Our study confirms that advanced glycation end products and their receptors are involved in the development of diabetes. This is evidenced by the correlation of AGEs and their receptors with common biomarkers of this disease (creatinine, GFR, glycated hemogobin). AGEs contribute also to the development of cardiovascular diseases, as evidenced by the relationship between the results obtained during the experiments and the blood parameters of patients (HDL cholesterol, uric acid) treated in the Diabetes Clinic. It is now believed that the end products of advanced glycation may be an important diagnostic marker due to their long stay in the body. Their determination may allow the disease progression and the effectiveness of the therapy to be monitored. It is also worthwhile to analyze other receptors for AGEs, continuing the research on more trials. Perhaps the work will contribute to revealing the knowledge about the potential role of AGE receptors in the pathomechanism of diabetes and will allow to estimate their diagnostic value. This work is also an introduction to the estimation of the diagnostic value of MAGE. These studies have the potential to contribute to faster diagnosis and significantly improve the effectiveness of the treatment of diabetes and other degenerative diseases.

## Materials and methods

The research material consists of 58 serum samples of patients from the Department of Angiology, Diabetes and Hypertension of Wroclaw Medical University (Poland). This work has been carried out in accordance with the Declaration of Helsinki (2000) of the World Medical Association. The research was approved by the Bioethics Committee of the Medical University in Wrocław (no. KB - 384/2012). Each participant of the study was familiarized with its purpose and signed an informed consent to participate in the study. Serum samples were collected from people between 26 and 82 years old, of whom 34 were female and 24 were male. Fifty of the patients had confirmed diabetes, 5 of them had type 1 diabetes, 44 type 2 diabetes, and 1 patient had type 3 diabetes (associated with Alzheimer's disease). In a clinical interview the information was obtained on the presence of arterial hypertension, atherosclerosis, hyperlipidemia, microangiopathy, macroangiopathy, nephropathy, polyneuropathy, ischemic stroke, ischemic heart disease and cigarette smoking. It is worth noting that only three of the patients had no hypertension. The results of patients blood parameters: HGB (hemoglobin), HbA1c (glycated hemoglobin), blood glucose, CRP (C reactive protein), TC (total cholesterol), LDL (low-density lipoproteins), HDL (high-density lipoproteins), TG (triglycerides), creatinine, GFR (glomerular filtratrion rate), c-peptide, RRs (systolic blood pressure), RRr (diastolic blood pressure) were analyzed. Information on the use of insulin therapy has also been obtained. The effect of taking cholinergic drugs, aspirin, clopidogrel, sulfonylureas, metformin and acabrosis was analyzed. All the results obtained in the experiments were compared with the above information on the patients health.

### Immunoenzymatic methods

#### Determination of MAGE

Competitive ELISA test antibodies and standards used herein are described in ^6^. 96-well MaxiSorp (Nunc) plates were covered with synthetic high molecular mass AGE (HMW-AGE) based on myoglobin, obtained by high temperature microwave synthesis. The coating process lasted 4 hours at a temperature of 37 °C. In the next step the plates were blocked with 10% skim milk for 18 h at 4 °C. Blood serum samples were dissolved in buffer containing proteinase K from Tritirachium album (Sigma Aldrich) with 30 U/mg activity (concentration of proteinase K in each serum was 0.3 mg/ml). The same samples of patient sera were also prepared for control purposes with only the buffer without proteinase K. The samples were mixed, centrifuged 1 min at 2000×*g* and incubated at 55 °C overnight in eppendorf tubes under parafilm. The samples were heated for 20 minutes at 116 °C to denature the proteinase, cooled and centrifuged for 15 min at 15,000×*g*, then diluted twice with PBS (without Ca2+ and Mg2+). The mixture was added to noncommercial monoclonal antibodies IgE anti-MAGE. 100 μl of the mixtures were applied to each well of Maxisorp plates and incubated at room temperature for 1.5 hours. In parallel, synthetic low molecular mass AGE (LMW-AGE) solutions were prepared for the preparation of a standard curve. The LMW-AGE was obtained by High Tempature Microwaves Synthesis and isolated by gel filtration on HW-40S and P2 bed. Plates were rinsed with PBS-T. A reaction was carried out with secondary anti-IgE antibodies (OriGene, polyclonal to mouse IgE- HRP; AP21482HR-N, working dilution 1: 7000) for 2.5 hours at room temperature, then plates were rinsed with PBS-T. The colorimetric reaction was developed by a solution containing 30 mg of

o-phenylenediamine (Sigma)/10 ml of PBS. Absorbance was measured at 450 nm in the EnSpire Manager microplate reader. The concentration of the protein in the serum samples was calculated based on the standard curve prepared on protein standard solution.

Intermediate ELISA test

Samples of diabetic patients sera and the LMW-AGE standard were prepared similarly to the above ELISA competition test. The plate was coated with serum solutions and LMW standard and incubated for 4 hours at room temperature. Then the plate was blocked with 5% skim milk in PBS-T overnight at 4 °C. The plates were washed with PBS-T and the primary anti-MAGE IgE antibodies (as above) diluted 2× with PBS were added to each well for incubation at room temperature for 3 h. After that, plate was washed with PBS-T and the secondary anti-IgE antibodies (OriGene, polyclonal to mouse IgE- HRP; AP21482HR-N, 1: 7000) were added to each well. After 1.5 hour incubation, the plate was washed and then the colometric reaction was developed using OPD (Sigma). The absorbance was read at 450 nm in an EnSpire Manager microplate reader.

#### Determination of sRAGE

sRAGE was determined using the ELISA kit for the sRAGE human receptor (MyBioSource, cat. no. MBS766075) according to the manufacturer's instructions.

#### Determination of SR-BI

Patients serum samples were diluted 10x and the standard SR-BI curve was prepared (Novus Biologicals, H00000949-P01) in the range of 0.009375 µg/100 µl–0.06 µg/100 µl. Coating was carried out overnight at 4 °C. The plate was blocked with 5% skim milk for 4 hours at 4 °C. Then, rabbit anti-SR-BI antibodies IgG (abcam, ab217318) at a dilution of 1: 1000 were used for 3 hours at room temperature. The secondary antibodies goat anti-rabbit IgG conjugate at a dilution of 1: 10.000 (Novus Biologicals, NB7187) were incubated 1.5 hours at room temperature. After reaction with the OPD substrate, the absorbance at 450 nm was read in an Epoch microplate reader.

#### Determination of SR-AI:

Serum samples were diluted 20x. A standard curve of SR-AI protein was also prepared (R&D, 2708-MS-050), in the range of 0.0025-0.5µg/100µl. Further steps were carried out as in the ELISA assay as above for the SR-B1 determination. We used goat polyclonal primary anti-SR-AI antibodies IgG, at a dilution of 1:50,000 (R&D, AF2708). After a 3-hour incubation, donkey anti-goat secondary antibodies IgG at a dilution of 1: 2000 (abcam, ab6885) were applied. After 1.5 hour incubation, the reaction with the OPD substrate was performed and absorbance at 450 nm was read with the Epoch microplate reader.

### Determination of total AGEs and low molecular mass AGEs (pentosidine) by means of fluorescence measurements

The methodology was adapted from the work of Leszek et al ^54^.

#### Fluorescence of total AGE and pentosidine

Samples of patients sera were diluted 1000 times with 0.9% NaCl and applied at quartz plate. Absorbance was measured in a SpectraMax® plate reader at 280 nm. Then diluted serum was applied to the wells of the plastic black plate (NuncTM). To determine the total amount of AGE in the serum, the fluorescence was measured by the excitation at wavelength 370 nm and emission at 440 nm. For measurement of pentosidine, excitation/emission wavelength was set at 335/385 nm. The amount of total AGE and pentosidine was expressed in arbitrary units [(F370 / 440): A280 and (F335 / 385): A280].

#### Fluorescence of protein-bound and low-molecular-mass AGEs

100 μl of undiluted sera from diabetic patients and 100 μl trichloroacetic acid (TCA) were mixed, and after 10 min of incubation, the samples were centrifuged at 5000xg for 20 min. The supernatant was separated and the precipitate was left to dry overnight. The supernatant was diluted 80x and its absorbance was checked at 280nm in a quartz plate. To determine the low molecular mass AGEs in the serum, the fluorescence of the supernatant samples in the black plate was measured at an excitation wavelength of 370 nm and emission 440 nm. For the measurement of pentosidine, excitation wavelength was set at 335, emission at 385 nm. The next day, 500 μl Tris-HCl buffer pH 7.4 was added to all tubes containing protein precipitate. The procedure for measuring absorbance at 280 nm on a quartz plate was repeated. To determine the total amount of AGE bound to proteins, the fluorescence was measured at excitation/emission wavelengths of 370/440 nm. For the measurement of pentosidine in the sediment, excitation and emission wavelengths were 335 and 385 nm.

### Statistics

Statistical evaluation of the obtained results was carried out using the GraphPad Prism 5 software. To obtain results on individual pairs of tests performed, the correlation coefficient r-Pearson and statistical significance (*p*) were calculated. Correlation graphs were created for statistically significant results. The assessment of dependence between individual blood parameters and experimental results, was performed in SPSS 24.0. The Levene test was used to determine if the variance was equal and the Student's t-test for independent tests. The average result for each trial was calculated and the groups were compared to be statistically significantly different (*p* <0.005).
